# Adaptive immune protection of the middle ears differs from that of the respiratory tract

**DOI:** 10.3389/fcimb.2023.1288057

**Published:** 2023-12-06

**Authors:** Kalyan K. Dewan, Amanda Caulfield, Yang Su, Colleen J. Sedney, Maiya Callender, Jillian Masters, Uriel Blas-Machado, Eric T. Harvill

**Affiliations:** ^1^ Department of Infectious Diseases, College of Veterinary Medicine, University of Georgia, Athens, GA, United States; ^2^ Department of Pathology, College of Veterinary Medicine, University of Georgia, Athens, GA, United States

**Keywords:** otitis media, adaptive immunity, *Bordetella bronchiseptica*, natural model, vaccination

## Abstract

The efficacy of the adaptive immune system in the middle ear (ME) is well established, but the mechanisms are not as well defined as those of gastrointestinal or respiratory tracts. While cellular elements of the adaptive response have been detected in the MEs following infections (or intranasal immunizations), their specific contributions to protecting the organ against reinfections are unknown. How immune protection mechanisms of the MEs compares with those in the adjacent and attached upper and lower respiratory airways remains unclear. To address these knowledge gaps, we used an established mouse respiratory infection model that we recently showed also involves ME infections. *Bordetella bronchiseptica* delivered to the external nares of mice in tiny numbers very efficiently infects the respiratory tract and ascends the Eustachian tube to colonize and infect the MEs, where it causes severe but acute inflammation resembling human acute otitis media (AOM). Since this AOM naturally resolves, we here examine the immunological mechanisms that clear infection and protect against subsequent infection, to guide efforts to induce protective immunity in the ME. Our results show that once the MEs are cleared of a primary *B. bronchiseptica* infection, the convalescent organ is strongly protected from reinfection by the pathogen despite its persistence in the upper respiratory tract, suggesting important immunological differences in these adjacent and connected organs. CD4+ and CD8+ T cells trafficked to the MEs following infection and were necessary to robustly protect against secondary challenge. Intranasal vaccination with heat killed *B. bronchiseptica* conferred robust protection against infection to the MEs, even though the nasopharynx itself was only partially protected. These data establish the MEs as discrete effector sites of adaptive immunity and shows that effective protection in the MEs and the respiratory tract is significantly different. This model system allows the dissection of immunological mechanisms that can prevent bacteria in the nasopharynx from ascending the ET to colonize the ME.

## Introduction

Certain pathogens and commensals of the upper respiratory airways can ascend the Eustachian tube (ET) to reach, colonize and infect the middle ears (MEs) (Bluestone et al., 1992; Soriano, 1997; Ngo et al., 2016). This often happens during transient disruptions of the innate immune barriers to the MEs caused by infections of the upper respiratory tract and is particularly prevalent among infants (Heikkinen, 2000; Chonmaitree et al., 2008). The resulting inflammatory response in the ME, otitis media (OM), involves pain and fever (Schilder et al., 2016) and can be traumatic for both patient and caregiver. Although most infections are acute (Acute Otitis Media: AOM) and resolve within a fortnight, the sheer volume of cases (Monasta et al., 2012; Schilder et al., 2016) make AOM amongst the leading causes of urgent pediatric clinic visits, ambulatory surgery and prescribing of antibiotics (Rosenfeld et al., 2022). In addition, the “proneness” to suffer OM varies (Alho et al., 1991; Pichichero, 2016), and in an estimated 8% of cases the infections can progress to several chronic forms of ear diseases (Chronic Otitis Media, COM) where the outcomes are more severe, and can lead to hearing impairment and deafness (Schilder et al., 2016; Bhutta et al., 2017). The societal impact of preventing OM would indeed be profound. However, controlling the burden of disease using vaccines targeted against upper respiratory tract pathogens remains challenging due to our relatively poor understanding of adaptive immunity in the ME (Bluestone et al., 1992; Soriano, 1997; Bakaletz, 2010; Ngo et al., 2016; Pichichero et al., 2023). There are limitations to OM infection models using human otopathogens, which alone do not generally infect the MEs of healthy mice, limiting our ability to study and understand the generation of effective acquired immune protection in the ME. A particular knowledge gap lies in understanding how immune components might contribute differently to protection of the ME and the connected respiratory airways (Kadioglu et al., 2002; Cho et al., 2021; Zohar et al., 2022).

Over the course of conducting studies on the respiratory tract pathogens belonging to the genus *Bordetella* (Linz et al., 2019), we observed and reported that two mouse pathogens, *Bordetella pseudohinzii* (Dewan et al., 2019) and *Bordetella bronchiseptica* (Dewan et al., 2022) can rapidly and efficiently ascend the ET from the nasopharynx to colonize and infect the MEs of mice. While infections by *B. pseudohinzii* are chronic and persist in the MEs, eventually leading to the deafness of the mice, *B. bronchiseptica* is controlled and subsequently cleared from the MEs over a period of convalescence coincident with the generation of a robust immune response. Here we use the *B. bronchiseptica*-mouse infection model in which bacteria in the nasopharynx naturally and efficiently ascend the ET to colonize and transiently infect the ME but are cleared by adaptive immunity. We identify the immune parameters induced by infection and vaccination and define their roles in protecting the ME, and how those roles differ from that of the adjacent and contiguous respiratory organs.

## Materials and methods

### Bacterial cultures

*Bordetella bronchiseptica RB50* and its gentamicin derivative resistant strain were maintained on Bordet-Gengou agar (DIFCO) supplemented with 10% sheep blood (Hema Resources) and 20 μg/mL streptomycin or gentamicin (Sigma) incubated for 36 hours at 37°C. Liquid cultures of the bacteria for inoculations were prepared in antibiotic-supplemented Stainer-Scholte broth at 37°C with shaking (200 RPM). Bacterial numbers were estimated spectrophotometrically at OD of 600 nm (0.1 ≅ 2 x10^8^ CFU/mL).

Heat-killed bacteria used for intranasal vaccination was prepared by heating the bacteria (~ 1 x10^9^ CFU/mL) at 65^0^C for 45 minutes in PBS. Aliquots of the samples were plated on BG agar to confirm loss of bacterial viability following treatment. Mice were immunized with 50μL of heat killed bacteria (5x10^7^ CFU).

### Mouse infections

The mouse strains, C57BL/6J, and derivative CD45.1 (B6.SJL-*Ptprca Pepcb*/BoyJ) and the *Rag-1^-/-^*, used in this study were obtained from Jackson laboratories and housed in SPF facilities at the University of Georgia throughout the experiments. Experiments were conducted with an n= 3-5 mice. Low dose nasopharyngeal infections were initiated by delivering ~ 500 CFU of the bacteria in 5 μL of PBS onto the external nares of lightly anesthetized mice (5% isoflurane). High dose bacterial challenges were conducted by droplet inhalation of ~ 5 x 10^5^ CFU of the bacteria suspended in 50 μL of PBS and delivered onto the external nares of anesthetized mice. To assess the colonization load, respiratory organs (nasal cavities, trachea lungs and middle ears) were carefully dissected out from mice that had been euthanized by CO_2_ inhalation (1.5 L/min). For establishing bacterial loads, the organs were homogenized in a bead mill beater 1 mL of PBS and CFU numbers enumerated by dilution plating aliquots of the homogenized samples on Bordet-Gengou agar with appropriate antibiotics. Where flow cytometric analysis was conducted, organs were collected in ice-cold DMEM and carefully ground over 70-micron filter. Cellular content of the filtrate was gathered by centrifugation at 4000g for 5 minutes at 4^0^C and resuspended in Dulbecco modified Eagle medium (DMEM) for subsequent flow analyses.

### Flow cytometry

Nasal cavities (NALT), lungs, and/or middle ears were processed and stained as previously described. Viable cells were identified with Zombie Aqua (Biolegend). Antibodies to identify T cell populations were (Biolegend, Invitrogen) included CD45.2 (AF 700), CD45.1 (PE Dazzle 594), CD3 (APC), CD4 (VF450), CD8 (APC Fire 750). Acquisition of the data was performed using the Novocyte Quanteon (Agilent) and analyzed with NovoExpress software (Agilent) following standard gating strategies (**Supplementary Figures 4**, **5**).

### Histology

Histopathology of the middle ears was assessed (n= 4 - 5) following fixation of mice in neutral-buffered, 10% formalin solution and subsequent decalcification in Kristensen’s solution. Tissues were embedded in paraffin, sectioned at approximately 5 μm. Coronal sections through the nose and brain, and transverse sections at the level of the middle and inner ear were collected and stained with hematoxylin and eosin. Histopathological exam consisted of evaluation of the nose for the incidence (presence or absence), severity, and distribution of inflammation.

### Statistical analysis

Data generated was statistically evaluated by Student’s t-test and 2-way ANOVA using the statistical analyses package of GraphPad prism (V2.0).

## Results

### *B. bronchiseptica* follows a course of primary infection in the middle ears distinct from that of the upper and lower respiratory tract

**Figure 1:**
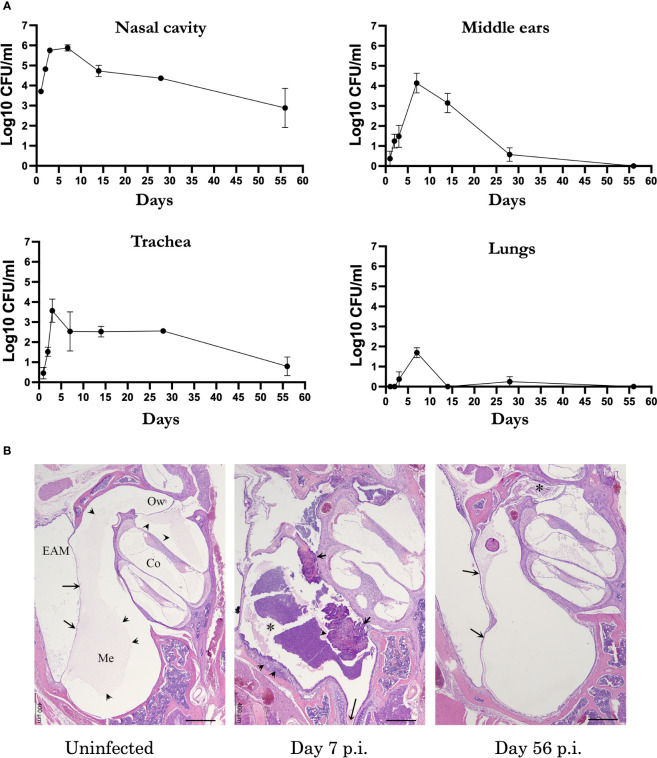
*B*. *bronchispetica* causes acute middle ear infections in mice. **(A)** Graph shows the CFU numbers of *B*. *bronchiseptica* recovered from the respiratory tract (nasal cavities, trachea and lungs) and middle ears of C57BL/6J mice over 56 days p.i. Dotted line represent the limit of detection (n=5, experiment conducted 2 times). **(B)** Representative Hematoxylin and eosin (HE) stained sections of the middle ears of uninfected (left panel), 7 days (central panel) and 56 days post inoculated mice(n = 4 mice). Left panel: Section of the middle ear from an uninfected mouse. Arrows point to the tympanic membrane. Within the middle ear (Me) and cochlea (Co), there is a pale eosinophilic (proteinaceous) material (arrowheads). Bar = 400 μm. Central panel: At the peak of infection on day 7, an inflammatory cellular infiltrate fills the middle ear (*) and expands the hyperplastic mucoperiosteum lining the inferior tympanic bulla (arrowheads). The long arrow points to the Eustachian tube. Right panel: By day 56 p.i., the degree of inflammation has subsided. In the current image, there is a small amounts of inflammatory infiltrate mixed with cholesterol clefts obscure the oval window (*). Arrows point to the tympanic membrane. Abbreviations: EAM, External Auditory Meatus; Ow, Oval window.

Respiratory pathogens associated with AOM in humans can often be detected persisting within the nasopharynx after OM resolves (Weiser, 2010; Aebi, 2011; King, 2012), suggesting immune responses may function more robustly and/or effectively in the ME. To define how an infection of the MEs proceeds, and how it might differ from that of the respiratory tract, we used *B. bronchiseptica* to follow the course of infection in groups of wild type (C57BL/6J) mice. The mice were inoculated intranasally with a low number of *B. bronchiseptica* in a small volume of PBS (~500 CFU in 5µL PBS) to localize the initial infection to the nasal cavities. Bacterial load in respiratory organs (nasal cavities, trachea, lungs) and MEs were then monitored over days 1, 2, 3, 7, 14, 28 and 56 post inoculation (p.i.) (**Figure 1A**). As expected, *B. bronchiseptica* grew rapidly (~1000-fold) in the nasal cavity to very high numbers (>100,000 cfu) in the first week, gradually declining thereafter but persisting for 8 weeks here, and for life in every mouse in many prior experiments, as expected for a highly efficient nasopharyngeal commensal/pathogen. Also as expected, *B. bronchiseptica* remained mostly undetected in the lungs (limit of detection 10 CFU). However, although *B. bronchiseptica* was not initially delivered to the ME, both MEs of all animals were colonized by day 2 p.i. and high numbers (~10,000 CFU) were recovered at day 7 p.i. These numbers then dropped close to detection limits by day 28 p.i. and no bacteria were recovered from the MEs by day 56 p.i., indicating complete clearance. Histological examination of HE stained sections from previous studies (Dewan et al., 2022) showed evidence of inflammation and hypercellularity at day 7 p.i (peak of infection) (**Figure 1B**). Here we find that by day 56 inflammation had subsided. These results show that despite being contiguous, the course of infection of the MEs differs from that of either the upper or the lower respiratory tract. The results also show that *B. bronchiseptica*, generally considered a respiratory pathogen, can rapidly overcome the substantial physical (cilia and mucus entrapment) and immunological (various innate responses) barriers of the Eustachian tube to efficiently reach and multiply within the MEs but does not effectively reach the lower respiratory tract. This is an important concern for conventional experimental studies of *Bordetella* spp. virulence mechanisms, which generally involve delivering *B. bronchiseptica* deep into the lungs, as discussed below.

### MEs of convalescent mice are robustly protected against secondary challenge

The observation that *B. bronchiseptica* efficiently colonized the ME but is cleared over the space of about four weeks implicates adaptive immune functions in providing sterilizing immunity in the ME. To determine whether mice that cleared *B. bronchiseptica* from the ME have effective anamnestic immunity, and contrast immunity in the ME to that in respiratory organs, we tested how well convalescent mice responded to a secondary challenge. We inoculated mice (n=5) with the low dose *B. bronchiseptica* as above and allowed the infection to progress for 56 days. On day 56 p.i, the convalescent mice and an age- matched naïve group, were challenged with the conventional high dose-high volume inoculum (5x10^5^ CFU delivered in 50 μL PBS) of a gentamicin resistant derivative of wild type *B. bronchiseptica*, to distinguish the very high dose/volume secondary challenge from remnants of the initial low dose/volume inoculation (primary challenge). The strength of the high dose and volume challenge is that it ensures that the entire respiratory tract receives a substantial challenging load of the pathogen, although its delivery of bacteria to the ME is less well known. 3 days post challenge mice were sacrificed and bacterial loads in the respiratory organs and MEs were enumerated. The low dose primary (genatamicin-sensitive) and high dose challenge (gentamicin-resistant) bacteria were differentiated based on their ability to grow on the gentamicin supplemented agar plates.

**Figure 2:**
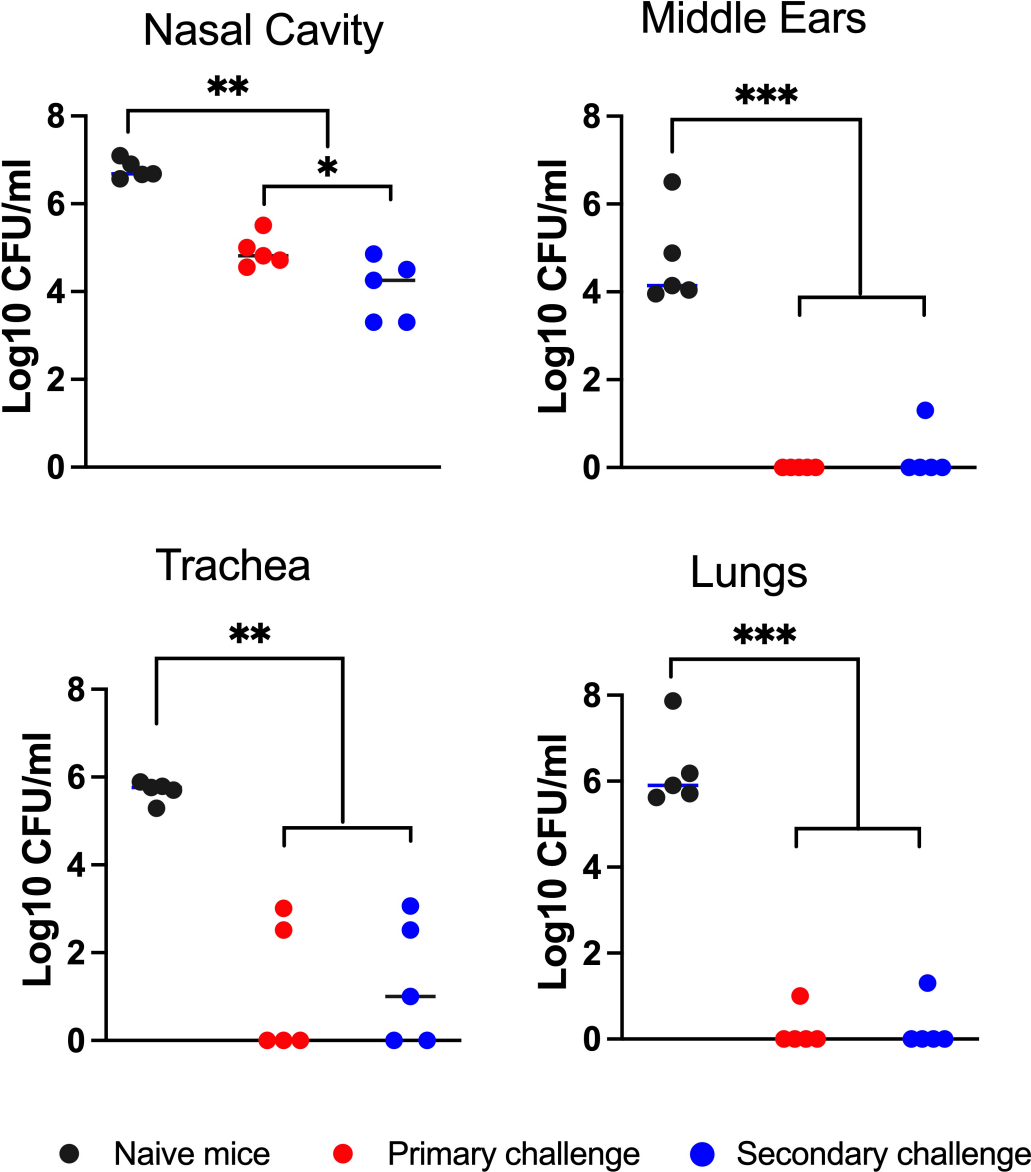
Robust convalescent immune protection in the middle ears. Graph shows bacterial CFU recovered from the nasal cavity, middle ears, trachea and lungs of mice after they had been intransally inoculated (1^0^ challenge) with 500 CFU (5μL of PBS) of *B*. *bronchiseptica* and re-challenged (2^0^ challenge) 56 days later with 5x10^5^ CFU (50 μL of PBS) of a gentamicin resistant isogenic strain of the bacteria. The bacterial CFU shown are from 3 days following the 2^0^ challenge. The number of gentamicin-resistant CFU (red circles) and gentamicin-sensitive CFU (blue circles) are independently displayed to distinguish the bacterial populations between the 1^0^ and 2^0^ challenges. Dashed line represents limit of detection. Statistical analysis was calculated *via* one-way ANOVA. [*P> 0.05; **P> 0.001; ***P> 0.0001] (n=5, experiment conducted twice).

As shown in **Figure 2**, the naïve control group had high numbers of CFU in all respiratory organs and MEs (between 10,000 and 1,000,000 CFU). The convalescent group retained relatively small numbers of the primary challenge inocula in the nasal cavity and trachea but had nearly or completely cleared this from the MEs and lungs. Following the secondary challenge, bacterial numbers in the nasal cavities of these convalescent mice were reduced by 100-fold relative to naïve mice, indicating partial but incomplete protection. In the lungs, robust protection was observed against the secondary challenge, demonstrating effective protective immunity despite almost no detected bacteria colonizing the lower respiratory tract over the course of the initial 56 day infection (**Figure 1A**). Interestingly, *B. bronchiseptica* was nearly or completely cleared from the ME, reflecting a level of protection similar to that of the lungs, and substantially more complete than that of the nasal cavity. We also observed similar protection when convalescent mice were rechallenged with low numbers of *B. bronchiseptica* (500 CFU delivered in 5 μL PBS) (**Supplementary Figure 1**). These results demonstrate that the ME of convalescent mice are robustly protected from reinfection and show that adaptive immunity in the MEs is as effective as that of the lower respiratory tract, and significantly more protective than that of the nasal cavity.

### Immune cells from the spleens traffic to the middle ears

We next turned our attention to examining the contributions of humoral and cellular components of adaptive immunity on convalescent protection. We had previously observed (Dewan et al., 2022) that when immune serum collected from convalescent WT mice (day 56 p.i.) was intraperitoneally injected into naive *Rag-1^-/-^* mice and challenged, bacterial CFUs in the MEs were reduced by ~ 100-fold compared to untreated *Rag-1^-/-^* mice. This indicated that serum IgG antibodies can substantially protect the MEs, though serum antibodies alone do not account for the near sterilizing immunity seen in the MEs of convalescent WT mice. To examine the contribution of cellular immunity to protection of the MEs, we examined whether immune cells from secondary lymphoid organs (SLOs) would traffic to the MEs in response to infections. We prepared single cell suspensions from spleens isolated from naive 6-7 week old C57BL/6J- CD45.1 (B6.SJL-*Ptprca Pepcb*/BoyJ) mice and adoptively transferred them into *Rag-1* ^-/-^ mice, which lack mature T- and B-cells. 3 hours after this transfer we intranasally inoculated the treated mice with a low dose inoculum of *B. bronchiseptica* (500 CFU/5µL PBS) to initiate nasopharyngeal infections. As controls we included a group of splenocyte transferred, but uninfected *Rag-1* ^-/-^ mice.

**Figure 3:**
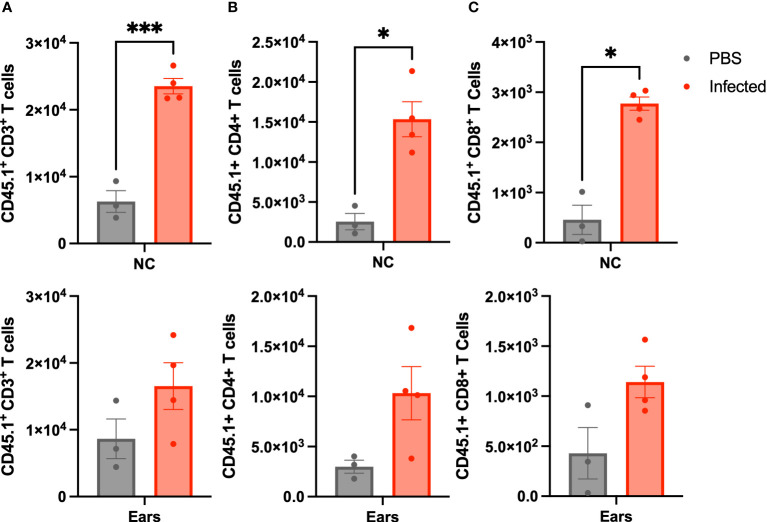
Lymphoid cells traffic to the ME in response to infection. Graphs show the number of CD45.1 marked **(A)** CD3^+^, **(B)** CD4^+^(b), and CD8^+^ **(C)**, cells isolated from *Rag-1* ^-/-^ mice that had been adoptively transferred with splenocytes from C57BL/6J (CD45.1) mice that were either left uninfected (grey-left columns), or inoculated 3 hours later with 500 CFU of *B*. *bronchiseptica* delivered in 5 μL of PBS (red-right columns). All mice were examined at 21 d.p.i. and cells recovered and enumerated from nasal cavity (NC) and ME (n=4 infected, 3 uninfected, experiment conducted once). Statistical analysis was calculated *via* one-way ANOVA. [*p<0.05, ***p<0.001].

At day 21 p.i, we sacrificed the mice and enumerated lymphocytes trafficked to the nasal cavities (NC) and MEs by flow cytometry. *Rag-1* ^-/-^ mice not given wild type spleen cells succumb to infection before day 21, so could not be included for comparison. As shown in **Figure 3**, both CD4^+^ and CD8^+^ T cells CD45.1 (confirming donor origin) T-cells were detected in the nasal cavities and ME samples of uninfected control *Rag-1* ^-/-^ mice. *B. bronchiseptica* infection induced > 2-fold increase in numbers of CD4^+^ and CD8^+^ cells in both the nasal cavities and the MEs, demonstrating similar recruitment and/or expansion of lymphoid cells in response to infections at these sites.

### Recruited T-cells complement the intraepithelial mucosa of the ME

**Figure 4:**
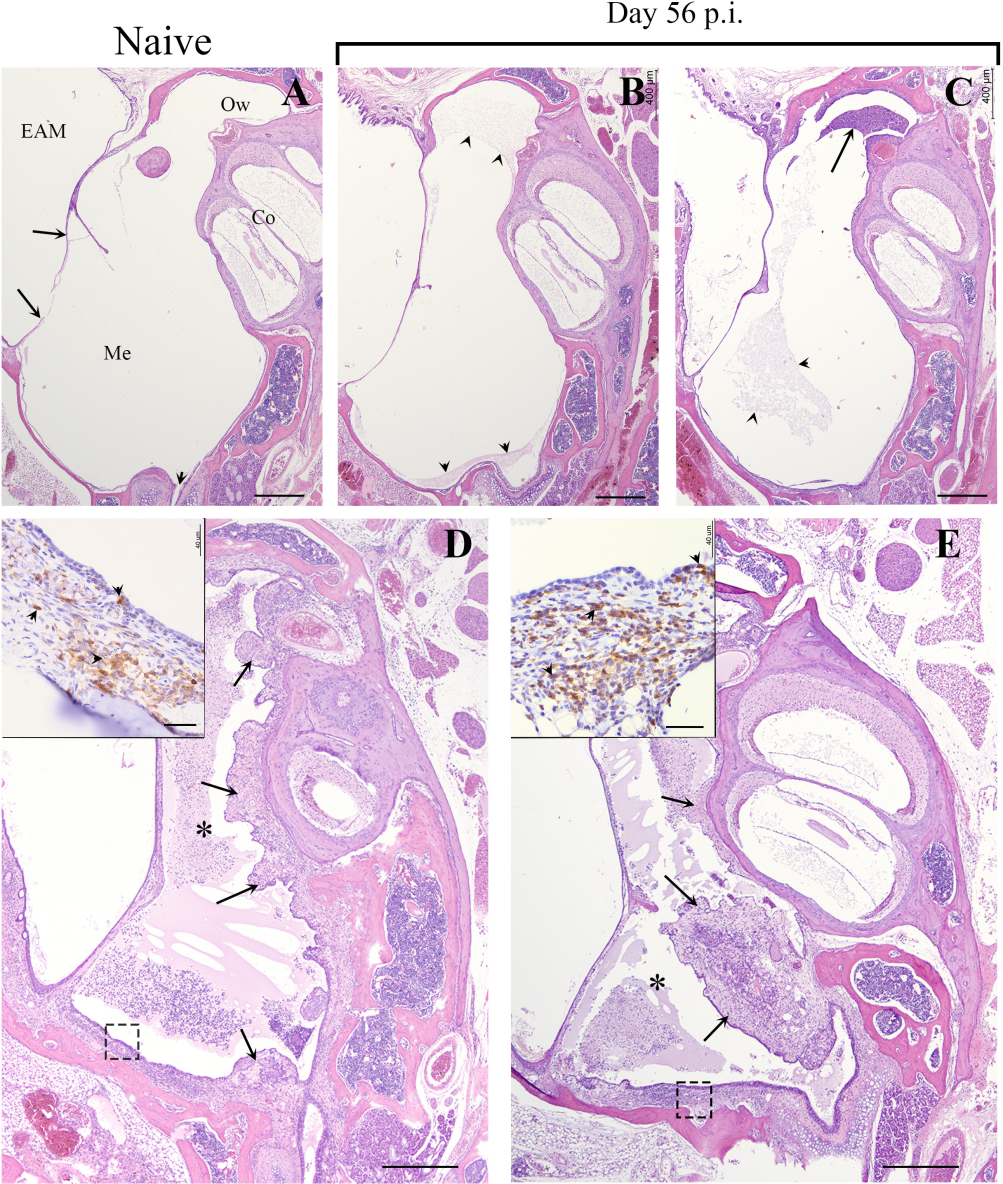
*B*. *bronchiseptica* middle ear infections in *Rag-1* ^-/-^ mice. Representative hematoxylin and eosin (HE) stained sections from the middle ears of uninfected/naïve **(A)** and 56 days post inoculated (p.i.) *Rag-1* ^-/-^ mice **(B–E)**. *Top panel*, **(A)** Section of the middle ear from an uninfected C57BL/6J mouse. Long arrows point to the tympanic membrane. Short arrow points to the Eustachian tube. Bar = 400 μm. *Top panel*, **(B, C)** Representative sections of the middle ear from two infected Rag-1^-/-^ mice with transferred splenocytes that were inoculated with 500 CFU of *B*. *bronchiseptica* and euthanized on Day 56 p.i. There is minimal accumulation of eosinophilic proteinaceous material (arrowheads) or inflammatory cellular infiltrate (arrow) within the middle ear of both mice. *Bottom panel*, **(D, E)** Representative sections of the middle ear from two inoculated *Rag-1* ^-/-^ mice with transferred splenocytes, euthanized on Day 56. Moderate to marked inflammatory cellular infiltrates embedded in eosinophilic proteinaceous material obscure most of the middle ear both mice (*, asterisks). In both mice, the proliferative, irregularly thickened mucoperiosteum is hypercellular (arrows). *Insets*: Higher magnification (Bar = 40 μm) of the mucoperiosteum outlined by a dash box with many CD3+ lymphocytes (brown; arrowheads), within the lamina propria and lining mucosa (arrows). Hematoxylin counterstain. EAM, External Auditory Meatus; Me, Middle ear; Co, Cochlea; Ow, Oval window.

As mentioned above, even low dose, low volume *B. bronchiseptica* inoculations are lethal to *Rag-1* ^-/-^ mice within weeks, but adoptively transferring wild type splenocytes rescued them from this lethality (**Supplementary Figure 2**). Although the MEs of the *Rag-1* ^-/-^ mice receiving wild type splenocytes did not clear the primary infections even at day 56 p.i. (**Supplementary Figure 3**), they allowed long term experiments and observations to be made. To examine where the recruited T-cells localized in the convalescent MEs, we conducted a histological study of splenocyte-transferred and *B. bronchiseptica*-infected *Rag-1* ^-/-^ mice at day 56 p.i. As shown in **Figure 4** (and in **Figure 1B**), H&E-stained sections of the day 56 p.i. MEs showed no gross pathology, though limited cellular infiltrates were observed (4B and 4C).

To localize T-cells in the MEs, H/E stained sections prepared from of the same ME sample block were incubated with HRP-conjugated anti-CD3^+^ antibodies. Developed sections showed aggregates of stained T-cells dispersed in the mucosa (inset **Figures 4D, E**), clearly demonstrating that T-cells are recruited to the ME in response to *B. bronchiseptica* and occupy the intraepithelial regions of the ME mucosa.

### Recruited immune cells protect the ME against secondary challenge

**Figure 5:**
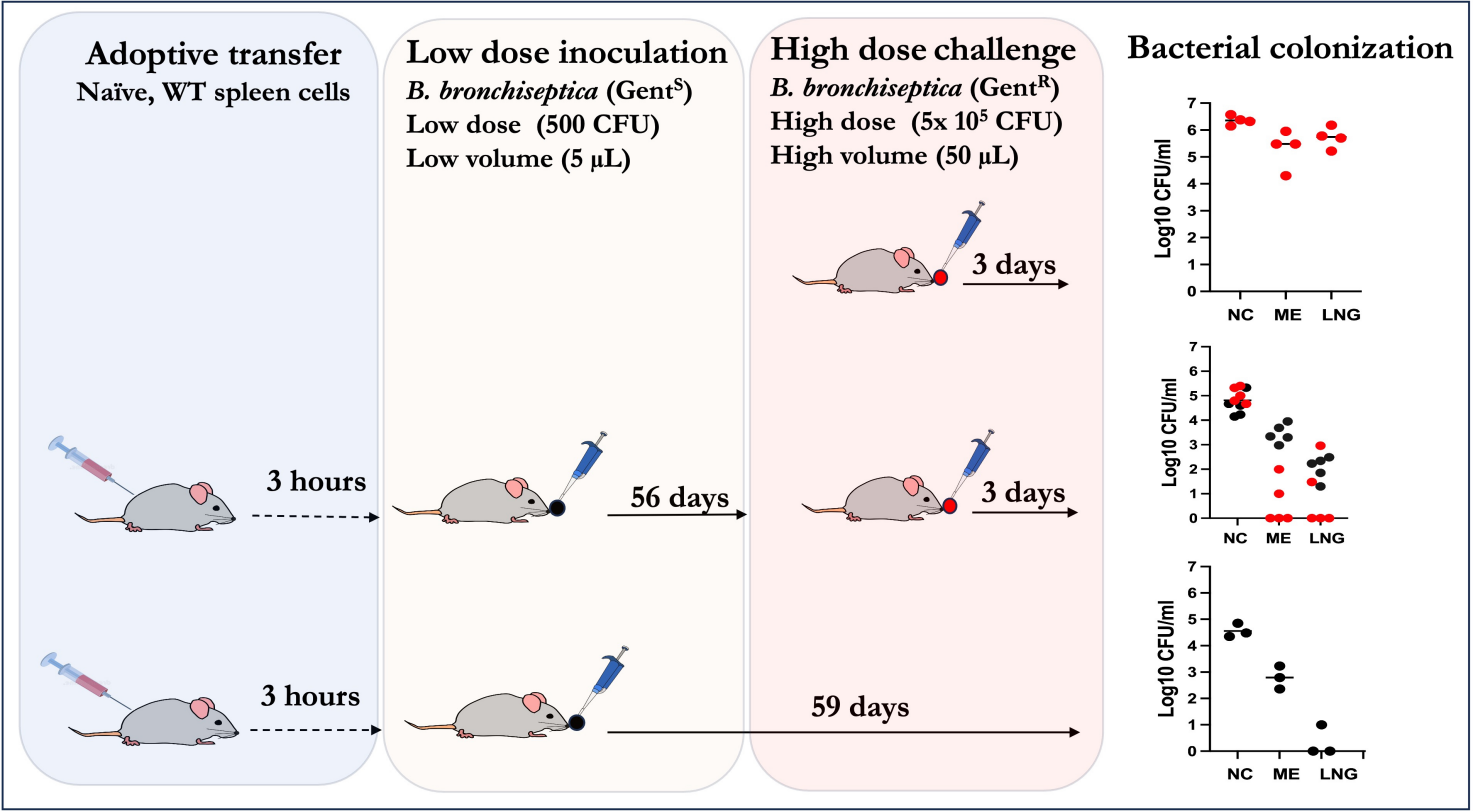
Schematic outline showing how adoptively transferred splenocytes are sufficient to confer robust convalescent protection to the MEs and lungs of *Rag-1^-/-^* mice from a high dose challenge of the bacterium. *Top panel*: Naïve *Rag-1* ^-/-^ mice challenged with 5x10^5^ CFU of a gentamicin derivative of *B*. *bronchiseptica* delivered in 50µL of PBS (*red* circles); *Middle panel*: *Rag-1* ^-/-^ mice given WT splenocytes, inoculated with low dose of *B*. *bronchisepica* RB50 (*black* circles) delivered in 5μL of PBS three hours later and then 56 days later challenged with 5x10^5^ CFU of the gentamicin derivative of *B.* *bronchiseptica* RB50 (as in top panel) delivered in 50μL of PBS and assessed three days later. *Bottom panel*: *Rag-1* ^-/-^ mice adoptively transferred (i.p.) with splenocytes three hours prior to primary challenge with 500 CFU of *B*. *bronchisepica* (*black* circles) delivered in 5μL of PBS three hours later. Colonization assessed 59 days later. Corresponding graphs on the right show the bacterial CFU recovered from the nasal cavities (NC), middle ears (ME) and lungs (LNG). (Experiments for each group were conducted >2 times).

We next examined whether recruited splenocytes protect the MEs of *Rag-1* ^-/-^ mice from secondary (high dose) challenges. As schematically outlined in **Figure 5**, splenocytes from naïve WT mice were adoptively transferred into two groups of *Rag-1* ^-/-^ mice (n=4) that were then inoculated with low dose inocula 3 hours later. On day 56 p.i. one group was challenged with the high dose inoculum of the gentamicin resistant *B. bronchiseptica* strain, while the second group (n=3) was left uninfected to determine the numbers of bacteria persisting from the primary low dose challenge (**Supplementary 3**). A third group of naïve *Rag-1* ^-/-^ mice were challenged with the high dose of the gentamicin resistant pathogen on day 56 p.i. to serve as non-protected (not transferred splenocytes) controls. After 3 days of the secondary high dose challenge, mice were sacrificed and bacterial loads in the nasal cavity, MEs and lungs were enumerated.

In the untreated (no splenocytes transferred) *Rag-1* ^-/-^ mice challenged with high dose inoculum (top panel), uniformly high bacterial CFUs were recovered from the nasal cavities (>1,000,000), MEs (>100,000) and lungs (>100,000). If left untreated these mice succumb to infection in about a month. In contrast, mice that received the splenocytes (middle panel) and a low dose of *B. bronchiseptica* 56 days earlier had moderate numbers (~100,000) in the nasal cavities. This (>10-fold reduction relative to the untreated mice suggests partial protection of the nasal cavities. In contrast, the bacterial numbers in MEs and lungs of splenocyte transferred and challenged mice were >99% reduced, relative to controls, indicating that, unlike in the nasopharynx, the transferred splenocytes were able to confer robust protective immunity. It is important to highlight that we still recovered gentamicin sensitive *B. bronchiseptica* in the nasal cavities and MEs (albeit only hundreds of CFUs in the lungs) indicating that the transferred/recruited splenocytes are by themselves not sufficient to completely clear primary ME infections. This is confirmed by the control mice (lower panel, n=3) receiving the splenocytes and the low dose inoculum. In this group, substantial bacterial CFUs were recovered from the nasal cavities (~10^5^ CFUs) while 100s of CFUs were also found in the MEs, but not in the lungs, indicating that the transferred/recruited splenocytes are by themselves sufficient to clear the lower respiratory tract, but are not sufficient to clear the ME. These results indicate that the anamnestic responses in the three contiguous compartments, i.e the MEs, lower and upper respiratory airways, are qualitatively distinct from each other. Furthermore, these data show that recruited immune cells from secondary lymphoid organs are insufficient to clear primary infections in the upper respiratory tracts but are necessary and sufficient to confer robust protection against subsequent challenges in the lungs and MEs.

### Intranasal immunization with heat-killed *B. bronchiseptica* robustly protects the MEs

**Figure 6:**
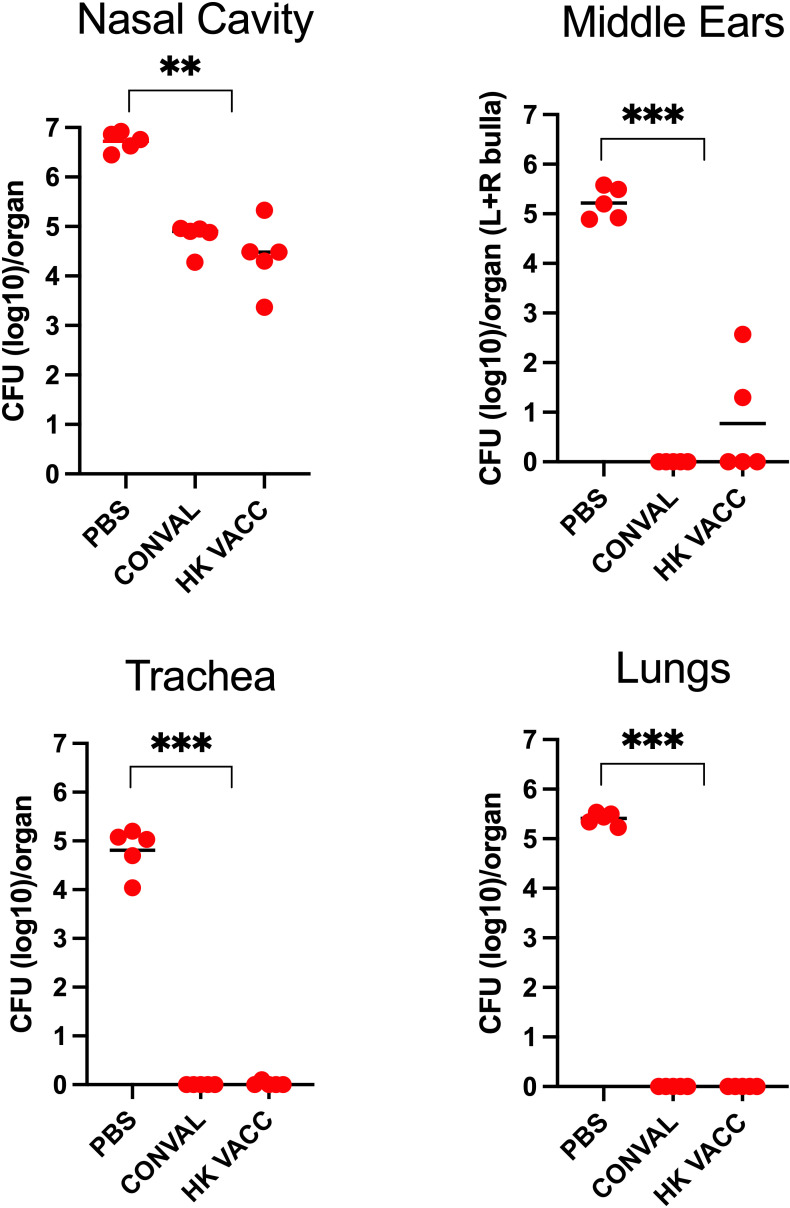
Graph shows bacterial CFU recovered from the nasal cavity, trachea, lungs and middle ears of naïve, convalescent, and vaccinated groups of C57BL/6J mice following a high dose challenge (5x10^5^ CFU in 50μL PBS) with *B*. *bronchiseptica* (circles) and analyzed 3 days later. Three groups of mice were examined: Naïve (PBS inoculated) mice, 56 d.p.i. convalescent mice following a low dose inoculum of *B*. *bronchiseptica* (500 CFU in 5μL of PBS), or mice intranasally vaccinated with 5x10^7^ CFU of heat killed *B*. *bronchiseptica*. (n=5; experiment conducted once). Statistical analysis was calculated *via* one-way ANOVA. [** p< 0.001; *** p< 0.0001].

We next examined whether vaccination induces similar protection in the MEs and respiratory organs. We used heat-killed whole cell *B. bronchiseptica* (5x10^7^ CFU/mL in 50μL of PBS) delivered intranasally, where mucosal stimulation against respiratory pathogens is known to be strong (Kurono, 2022) and is the recommended method of delivery for non-human animal vaccines to protect against *B. bronchiseptica* (Ellis et al., 2016; Ellis et al., 2017). To evaluate the levels of protection conferred by the vaccine relative to convalescent immune protection, we also included a group of mice that had been inoculated eight weeks earlier with a low dose of *B. bronchiseptica* (streptomycin resistant). Protection was assessed 3 days following high does challenge (**Figure 6**).

Naïve (PBS vaccinated) mice showed high bacterial loads in the MEs and respiratory organs. The convalescent group of mice showed strong adaptive immune protection in the MEs and lower respiratory tract while the nasal cavities were only partially protected. Interestingly, protection conferred by the intranasal vaccinations was very similar to that of the convalescent mice, successfully protecting the MEs and lower respiratory tract from high-dose challenge of *B. bronchiseptic*a. However, the nasal cavities remained only partially protected, indicating that immune mechanisms more efficiently protect the MEs than they do the upper respiratory tract, despite being the actual site of vaccination.

### Differential T-cell responses generated in middle ears relative to respiratory tract

**Figure 7:**
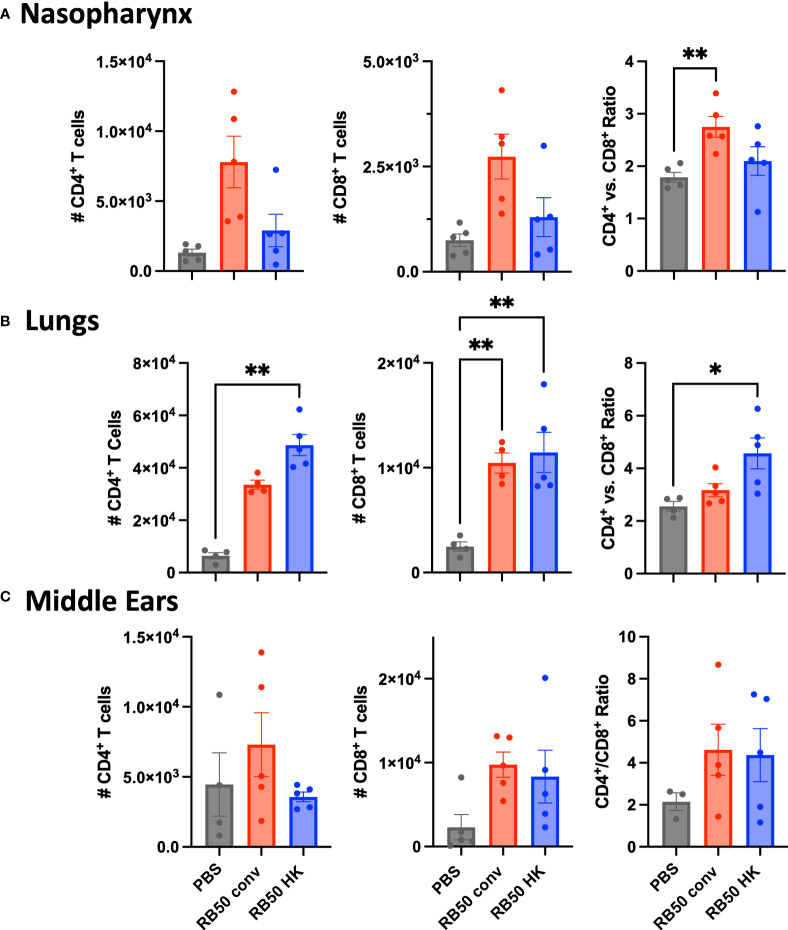
Total CD4^+^ and CD8^+^ T cells recovered from the mice in **Figure 6**. **(A)** nasal cavities, **(B)** lungs, and **(C)** middle ears of naïve, RB50-convalescent, or RB50-vaccinated C57BL6/J mice following high dose challenge (5X10^5^ CFU in 50μl PBS). Naïve (PBS inoculated) mice, 56 d.p.i. convalescent mice following a low dose inoculum of *B*. *bronchiseptica* (500 CFU in 5μL of PBS), or mice intranasally vaccinated with 5x10^7^ CFU of heat killed *B*. *bronchiseptica*. Statistical analysis was calculated *via* one-way ANOVA. Error bars show standard error of the mean (n= 5, experiment conducted once). [*p<0.05 and **p<0.002].

Flow cytometric analysis conducted on cells prepared from nasal, lung and ME samples from the above experiment did not indicate any expansion of B-cell populations on challenge in either the convalescent or vaccinated groups (**Supplementary Figure 6**). This has precluded any definitive conclusion on their contributions. However, we observed greater numbers of CD4^+^ and CD8^+^ T cells recovered from these immune animals relative to naïve mice in all these organs (**Figure 7**). Interestingly, though higher numbers of CD4^+^ T cells and differential CD4^+^/CD8^+^ ratios correlated with robust protection in the MEs (**Figures 7C**) and lungs (**Figure 7A**) of immune mice, these appear to be insufficient to facilitate clearance of the nasal cavity (**Figure 7B**) by 3 days post-challenge. This further supports the observation that robust protective T cell-mediated immunity in the MEs and lungs can be generated *via* upper respiratory infections *and* vaccination, yet the level of protection generated by either means is less complete in the nasopharynx.

## Discussion

Despite a compelling need to understand acquired immunity within the MEs, this has been particularly difficult to study using human otopathogens owing to their inability to naturally colonize the MEs of experimental mice that can be manipulated immunologically. A natural respiratory/middle ear infection model in mice is especially relevant to the study and understanding of adaptive immunity of the MEs given the connection of the organ, via the ET, to the upper respiratory airways from which most ME infections originate and where the early stages of cognate immunity in a naïve host are likely to be established. The mammalian upper respiratory tract commensal/pathogen, *B. bronchiseptica*, appears to be a natural otopathogen of mice, highly efficient in causing infections resembling human AOM. This experimental system is well-suited to investigate how AOM is cleared and how long-term immune protection of the MEs may be generated. As we have shown, *B. bronchiseptica* efficiently establishes inflammatory infections in the MEs of nasally inoculated mice that are eventually controlled and subsequently cleared. As observed for the prevalent human otopathogens (Ellis et al., 2016; Ellis et al., 2017; Kurono, 2022), *B. bronchiseptica* is cleared from the ME but persists in the nasopharynx indefinitely, underscoring the particulars of the protective immunological environment that surveils and protects the MEs from potential otopathogens that persist in the nasopharynx. Our assessment of the levels of protection in convalescent mice showed near sterilizing anamnestic immunity in the MEs and lungs, but only partial protection of the nasal cavities, highlighting the distinct environment of the MEs compared to the nasopharynx.

Adoptively transferring naïve wild type splenocytes into *Rag-1^-/-^* mice allowed peripheral T-cells to be recruited to the MEs in response to infection. This influx of lymphocytes from secondary lymphoid organs was further confirmed by the histopathological detection of CD3^+^ populations of cells complementing the intraepithelial mucosa (or mucoperiosteum) of the infected mice at day 56 p.i.

Prior studies (Kodama et al., 1999; Kodama et al., 2000; Sabirov et al., 2001) established that the MEs exhibit the features of an effector site of adaptive immunity, with increase in antigen specific secretory IgA titers and Th2 cytokines on antigen restimulation. Intranasal immunization also partially reduced the bacterial burden of experimentally injected non-typeable *H. influenzae* in the MEs of BALB/c mice compared to uninfected controls. However how this control relates to infection in the nasopharynx was not possible to assess due to the need to directly introduce the pathogen into the ME (Sabirov et al., 2001). We see here that intranasal immunizations using heat-killed *B. bronchiseptica* provide strong protection to the MEs, but only partially protect the nasopharynx. These results clearly indicate that intranasal vaccine-induced protection of the MEs can be very effective even against a commensal/pathogen that is highly adapted to successfully colonize and persist in the upper respiratory airways and establishes that in resisting such an efficient colonizer the ME is an independent and vaccine-targetable organ.

This initial investigation of adaptive immunity of the MEs using a natural model of AOM in mice demonstrates an experimental system that can address several pressing questions. Where along the Eustachian tube-ME mucosal geography is protection conferred by antibodies and immune cells? How might protective immunity be established to protect against other otopathogens and/or polymicrobial infections? Do viral infections or allergic immune disturbances compromise induced protection to facilitate (re)infections of the MEs? How might deficiencies in immune signaling compromise ME protection in naïve or convalescent hosts? Basha and Pichero (2015). Finally, since “nothing in biology makes sense except in light of evolution”, it is worth considering what possible evolutionary advantages to a pathogen there might be in infecting the ME, which is essentially a “dead end” site, only connected to the outside world via the ET and nasopharynx. *Prima facie*, colonizing the ME does not appear to contribute to transmission to new hosts. Are pathogens of the upper respiratory tract exploiting the ME as a reservoir from which to reseed the respiratory tract (Hardison et al., 2018), from which they can then transmit to other hosts? Or is the ability to infect the ME not itself an adaptive trait, but simply a side-effect of adaptation to infect deep into the upper respiratory tract? Considering these evolutionary questions may help guide studies to deepen our understanding of the infectious process of AOM and the complex interactions with the immune system that protects the ME.

## Data availability statement

The original contributions presented in the study are included in the article/**Supplementary Material**. Further inquiries can be directed to the corresponding author.

## Ethics statement

The animal study was approved by Institutional Animal Care and Use Committee of the University of Georgia, Athens, Georgia, USA. The study was conducted in accordance with the local legislation and institutional requirements.

## Author contributions

KD: Conceptualization, Data curation, Formal Analysis, Investigation, Methodology, Supervision, Writing – original draft, Writing – review & editing. AC: Formal Analysis, Investigation, Methodology, Writing – review & editing. YS: Data curation, Investigation, Writing – review & editing. CS: Data curation, Formal Analysis, Investigation, Writing – review & editing. MC: Data curation, Investigation, Writing – review & editing. JM: Formal Analysis, Investigation, Writing – review & editing, Data curation. UB-M: Data curation, Methodology, Supervision, Formal analysis, Validation, Investigation, Visualization, Writing – review & editing. EH: Investigation, Methodology, Conceptualization, Formal Analysis, Funding acquisition, Project administration, Resources, Supervision, Visualization, Writing – original draft, Writing – review & editing.
