# Supplementary material for: Adaptive immune protection of the middle ears differs from that of the respiratory tract
Source: Front Cell Infect Microbiol. 2023 Dec 6;13:1288057. doi: 10.3389/fcimb.2023.1288057 (PMC10731285; doi:10.3389/fcimb.2023.1288057)
Supplement: Supplementary file 1 [file DataSheet_1.pdf]

SUPPLEMENTARY INFORMATION

Supplementary Figure 1

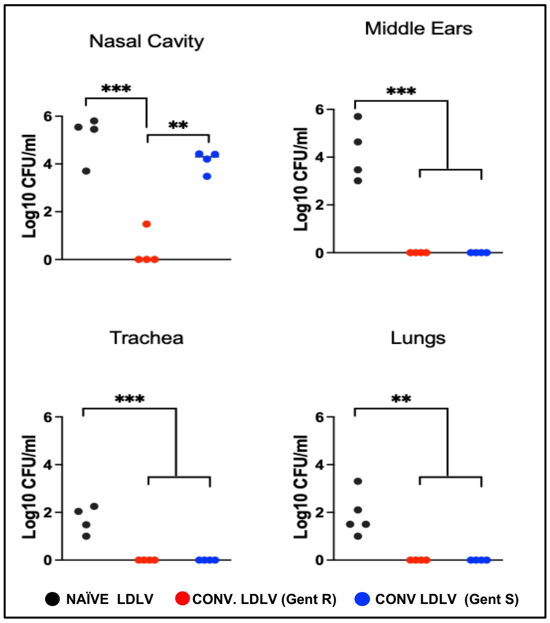

MEs of convalescent C57BL/6J mice protected against low dose low volume challenge of *B. bronchiseptica*

Graph shows bacterial CFU recovered from the nasal cavity, trachea, lungs and middle ears of C57BL/6J mice that had been intransally inoculated with 500 CFU (5uL of PBS) of *B. bronchiseptica* (gentamicin sensitive-blue) and challenged on day 56 p.i. with low dose low volume (LDLV) inoculum: 500 CFU of a gentamicin resitant (red) isogenic strain of the bacteria delivered intranasally in 5 uL of PBS. Control group LDLV challenged non-convalescent naïve mice (black circles). Mice were sacificed 7 days following this challenge. Statistical analysis was calcualted *via* one-way ANOVA. Error bars show standard error of the mean (n=4) (experiment conducted once).

Supplementary Figure 2

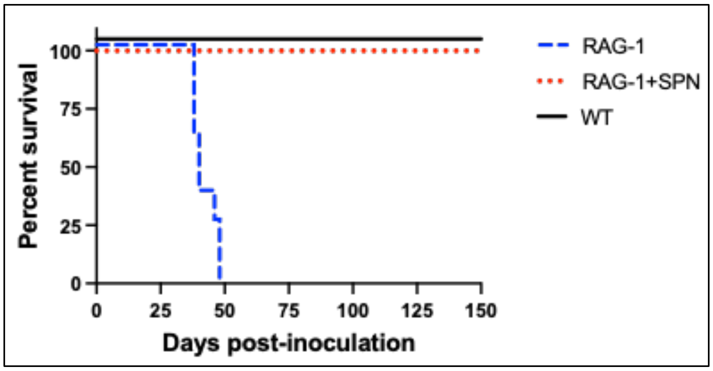

Adoptively transferred splenocytes protect *Rag-1*$^{-/-}$ mice against lethality from *B. bronchiseptica*

Kaplan-Meier survival curves of C57BL/6J (WT), *Rag-1*$^{-/-}$ mice (blue dashed line) and *Rag-1*$^{-/-}$ mice with adoptively transferred splenocytes from C57BL/6J (red dashed line) inoculated with *B. bronchiseptica* (500 CFU delivered in 5uL PBS). *B. bronchiseptica* infections are lethal to *Rag-1*$^{-/-}$ mice within 7 weeks. However, *Rag-1*$^{-/-}$ mice with WT splenocytes survive.

Supplementary Figure 3

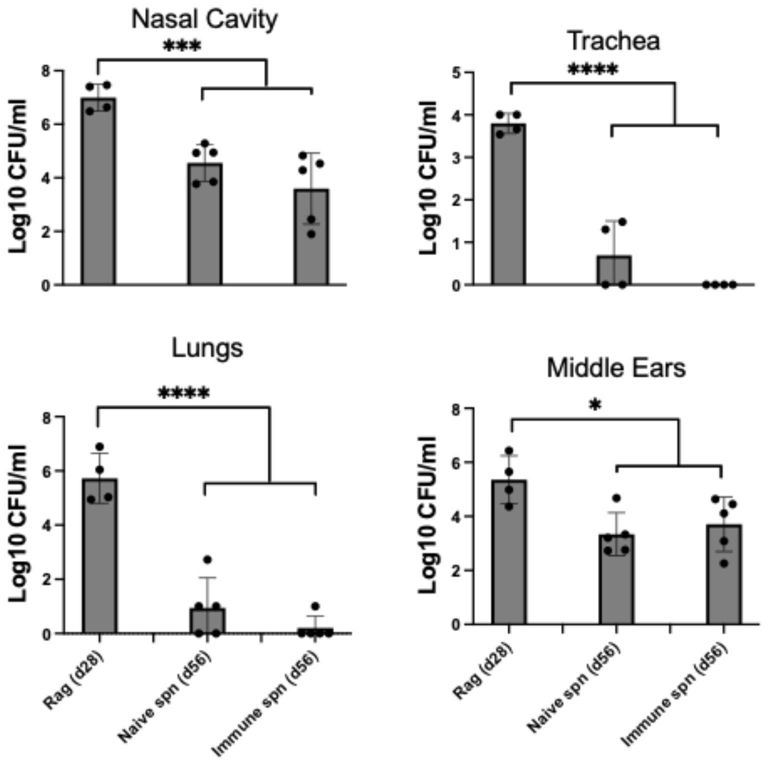

Adoptively transferred splenocytes protect *Rag-1$^{-/-}$* mice fail to clear *B. bronchiseptica* from the middle ears

Rag-1$^{-/-}$ mice with adoptively transferred splenocytes from either naïve (Naïve spn) or convalescent C57BL/6J mice (Immune spln) survived but, failed to clear *B. bronchiseptica* from the middle ears by day 56 p.i. when inoculated intranasally with 500 CFU suspended in 5uL PBS. (n=4 and 5) Statistical analysis was calcualted *via* one-way ANOVA. Error bars show standard error of the mean [p*<0.05, **<0.001, p****<0.0001] *Rag-1$^{-/-}$* mice without splenocyte transfers do not survive beyond 5-6 weeks and data is shown for day 28 p.i. Experiment conducted once.

Supplementary Figure 4

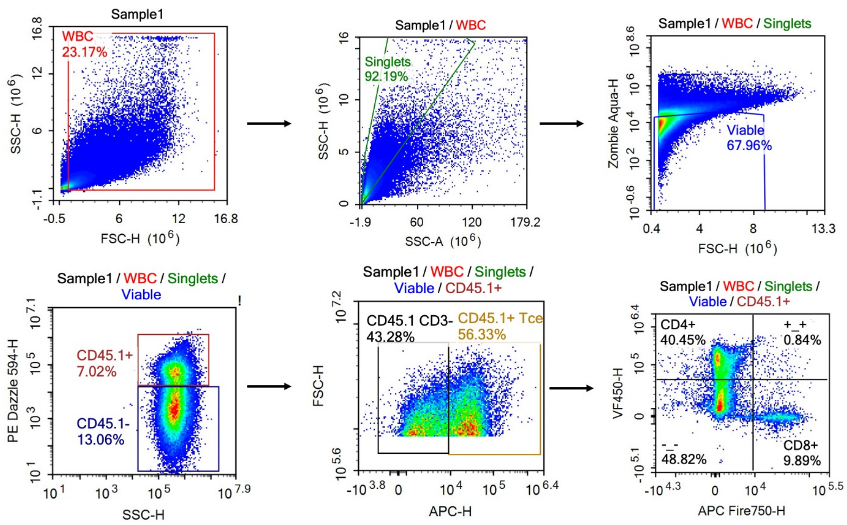

Gating strategy for the enumeration of transferred CD45.1^{+} CD4^{+}/CD8^{+} T cells trafficked to the middle ears of C57BL6/J mice.

Supplementary Figure 5

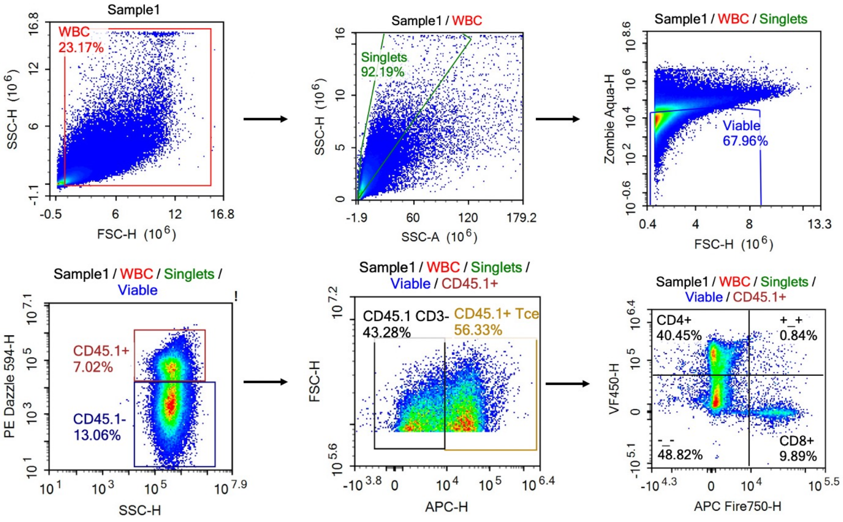

Gating strategy for the enumeration of transferred CD45.1^{+} CD4^{+}/CD8^{+} T cells trafficked to the middle ears of C57BL6/J mice.

Supplementary Figure 6

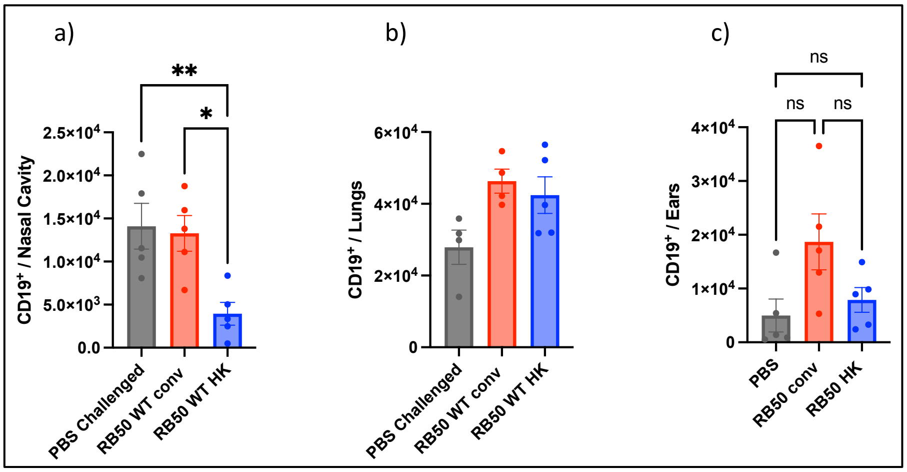

Total CD19+ cells recovered from the (a) nasal cavities, (b) lungs, and (c) middle ears of naïve, RB50-convalescent, or RB50 vaccinated C57BL6/J mice following high dose challenge ($5X10^5$ CFU in 50µl PBS). Naïve (PBS inoculated) mice, 56 d.p.i. convalescent mice following a low dose inoculum of *B. bronchiseptica* (500 CFU in 5µL of PBS), or mice intranasally vaccinated with $5x10^7$ CFU of heat killed *B. bronchiseptica*. Statistical analysis was calculated *via* one-way ANOVA. Error bars show standard error of the mean (n= 4-5). [*p<0.05 and **p<0.002].
